# Characteristics, reasons and patterns of Road Traffic Injuries presenting in emergency department of a tertiary care public hospital in Karachi

**DOI:** 10.12669/pjms.38.4.4490

**Published:** 2022

**Authors:** Khaista Muhammad, Shiraz Shaikh, Javeria Ashraf, Sikander Hayat

**Affiliations:** 1 Khaista Muhammad, BSN, MSPH, APPNA Institute of Public Health, Jinnah Sindh Medical University, Karachi, Pakistan; 2 Shiraz Shaikh, MBBS, FCPS. Associate Professor, APPNA Institute of Public Health, Jinnah Sindh Medical University, Karachi, Pakistan; 3 Javeria Ashraf, MBBS, FCPS. Assistant Professor, Jinnah Postgraduate Medical Centre, Karachi, Pakistan; 4 Sikander Hayat, MBBS, FCPS. Assistant Professor, Jinnah Postgraduate Medical Centre, Karachi, Pakistan

**Keywords:** RTIs (Road traffic injuries), RTAs (Road traffic accidents), Motorbike accidents

## Abstract

**Objectives:**

To determine the main characteristics, reasons and patterns of road traffic injuries (RTIs) in a tertiary care public hospital of Karachi.

**Methods:**

It was a hospital based cross sectional study conducted in a public tertiary care hospital emergency department with a sample size 425 selected conveniently. Participants included the consenting victims or caretaking attendants of road traffic injuries (RTIs) from 25^th^ May to 28^th^ June in 2019. A structured questionnaire was developed after literature review and was translated into “Urdu” language. The questionnaire collected detailed information on socio-demographic characteristics, possible reasons of RTI’s such as condition of vehicle, over speeding and breaking traffic rules. Data was analyzed by using software SPSS version 20.

**Results:**

Almost half of RTI victims (43.1%) belonged to the age group 18-29. Majority of the victims were males (86.6%). The most common vehicle involved was motorbike (87.50%) followed by Rickshaw (6.8%) and Car (2.4%). Majority of RTIs occurred on main road (75.30%). The most common sites of injuries were lower limb (64%), upper limb (37.60%) and head (32.2%). The severe injuries were significantly more likely to happen in events in which direct collision with other vehicle/thing was involved, road conditions were wet and pedestrian were crossing the road.

**Conclusion:**

Motorbikes were involved in majority of RTIs. Main reasons of RTIs included irresponsible road behaviors including over-speeding, careless road crossing, breaking the signal and riding on wet roads which lead to moderate to severe injuries in almost two thirds of participants

## INTRODUCTION

Road Traffic Injuries (RTIs) can occur as a result of a conjunction of human error, road-related conditions and vehicle defects.^1^,^2^ Globally, road traffic accidents account for approximately 1.27 million deaths worldwide per year, making them the 11th leading cause of death.^3^ Deaths due to RTIs are predicted to have declined by 27-30% in high-income countries.^4^ However, approximately 90% of deaths related to RTIs occur in low and middle income countries.^5^ The consequences of RTIs can range from minor injuries to disability and death, depending on their severity. Injuries and disabilities can further lead to psychological distress as well as loss of productivity.^6^-^8^

Due to rapid urbanization and motorization trends, traffic violations, road encroachment, and a lack of proper road safety programs RTI's are emerging as a serious public health concern in Pakistan. RTIs are estimated to occur 15 times per 1000 people per year, according to the National Injury Survey of Pakistan (NISP), 2004.^9^ About 11.3 people die per 10,000 vehicles registered in Karachi.^10^ Although a few studies have been conducted in different parts of Pakistan on RTIs, there is a lack of comprehensive data on their distribution, reasons and potential burden of disability.

The aim of this research was to contribute towards generating information on reasons and outcomes of RTIs which could help in identifying the priority interventions needed to prevent them. The main objectives were to evaluate the main RTI characteristics, reasons and injury patterns in a public sector tertiary care hospital in Karachi.

## METHODS

It was a cross-sectional hospital-based questionnaire study conducted in the emergency department of Jinnah Postgraduate Medical Center, Karachi. All patients presenting with RTIs regardless of the site of injury or severity were invited to participate in the study. Those who declined to give consent or were brought dead to the hospital were excluded. The research took six months to complete, from June to December 2019. Sample size was calculated using the software Open Epi. At a confidence level of 95%, precision of 5% and an anticipated proportion of different risk factors of RTIs ranging between 21%-76%, the maximum sample size obtained was 384 and was adjusted to 425. Due to short timeline and scope of the study, a non-probability convenient sampling technique was used to select the study participants.

After literature review and input from an emergency medical specialist, a structured questionnaire was finalized and translated in “Urdu” language, including detailed information on socio-demographic characteristics, main characteristics of RTI (site, time, type of vehicle involved, type of collision), possible reasons of RTIs related to the driver (over speeding, violating the traffic signal, smoking, intoxicated or drunk-drivers/riders, using the cell phone or chatting while driving, wearing inadequate entangling clothes especially on motorbikes, ignoring helmets), related to vehicle (mechanical fault, overloading), related to environment (wet and slippery roadways, shortage of streetlights in night timings, tactical error by the opposite rider/driver/pedestrian, unforeseeable road barriers and hurdles, reckless driving attitude) and severity of injury (mild, moderate and severe). Any injury leading to superficial cuts, scratches, scalp injury less than one centimeter in depth and length and sprain due to RTI was classified as mild. A moderate injury was the one that resulted in laceration with skin loss, damage to underline muscles, tissue, joint ligaments, and a scalp injury more than one cm in depth and less than 10 cm in length. Any injury resulting in a dislocation or fracture was considered a severe injury, whereas any injury resulting in permanent loss of mobility or function of any portion of the body is labeled as a disability. Ethical approval was taken from the Institutional Review Board at Jinnah Sindh Medical University (IRB/JSMU/2019/172). Informed consent was obtained from all participants. All the data was collected by the investigators.

Data was analyzed using software SPSS version 22. Descriptive statistics of variables were presented as mean, standard deviation or frequency percentages. Logistic regression was used to determine the effect of different factors i.e, age, education and different characteristics of RTIs on the occurrence of severe injuries. Factors that failed to meet the assumptions of being included in the multivariate model were excluded. Odds ratios with a 95% confidence interval were reported. A P-value of less than 0.05 was considered significant.

## RESULTS

Almost half of the victims (43.1%) were between the ages of 18 and 29, while the rest were evenly distributed among the different age groups (Table-I). Men comprised the majority of the victims (86.6%). Around one-fourth (25.1%) were illiterate and another one-fourth had only earned primary education (26.6%).

**Table-I:** Descriptive statistics of study participants (n=425).

***Age***	
4-17	15.8% (67)
18-29	43.1% (183)
30-39	20.2% (86)
40 and above	20.9% (89)
***Gender***	
Male	86.6% (368)
Female	13.4% (57)
***Education***	
Illiterate	25.2% (10 7)
Primary	26.1% (111)
Matriculate	29.4% (125)
Intermediate and above	19.1% (81)
Monthly Income	Mean = 18593.16
(n=263)	SD = 8707.71

The most common vehicles involved in RTIs were motorbikes (87.5%) followed by rickshaws (6.8%), cars (2.4%), trucks (1.6%) and others (1.6%). Table-II. Half of RTIs happened due to collisions between vehicles (52.70%) predominantly on the main roads (75.30%). During various times of the day, RTIs were almost evenly distributed.

**Table-II:** Characteristics of RTIs (n=425).

***Victims***	
Drivers/Riders	64% (272)
Others (Pedestrians/Person accompanying riders/drivers)	36% (153)
***Vehicle Involved in RTI***	
Motorbike	87.5% (372)
Rickshaw	6.8% (29)
Car	2.4% (10)
Truck	1.6% (7)
Others	1.6% (7)
***Nature of Accident***	
Collision with another vehicle	52.7% (224)
Collision with another object	11.1% (47)
Vehicle slippage	36.2% (154)
***Site of Accident***	
Main Road	75.3% (320)
Link Road	16.8% (72)
House Street	6.8% (29)
Round about	0.9% (4)
***Time of Accident***	
Night (21:00 to 8:00 hours).	30.4% (129)
Morning (8:00 to 12:00 hours).	26.4% (112)
Day (12:00 to 17:00 hours).	21.2% (90)
Evening (17:00 to 21:00 hours).	22.1% (94)
Motorbike riders with helmets (n=256)	12.5% (32)

**Fig.1:**
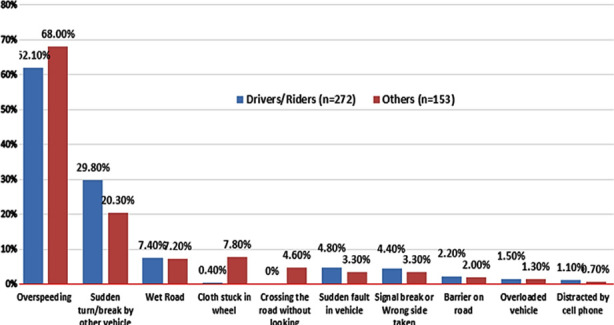
Reasons of RTIs among Victims n=425.

Reasons of RTIs among victims are shown in Fig.1. Majority of injuries among the drivers and riders were due over-speeding either by the vehicle of the driver and/or by another vehicle. Other common reasons were sudden break or turn by other vehicle, wet road and wrong side taken by another vehicle. Less common reasons were overloaded vehicle, barrier on road and distraction by talking on cell phone or looking at sign board. Among victims who were either pedestrians or persons accompanying riders and driver’s cloth stuck in wheel and crossing without looking were also common reasons.

More than half (54.6%) of the injuries were found moderate followed by mild (29.6%) and severe (15.8%). Few of the victims (6.4%) experienced disability. Relationship of different factors with severe injuries is shown in Table-III. In reference to RTI’s resulting due to slipping on road, those involved in collision with other vehicle/thing were significantly more likely (adjusted OR=3.16; 95% CI=1.53-6.51) to experience severe injuries. Among the risk factors included in the model, crossing without looking (adjusted OR=4.34; 95% CI=1.09-17.20) and wet road (adjusted OR=3.96; 95% CI=1.41-11.09) showed a significant positive association with the outcome.

**Table-III:** Relationship of socio-demographic and RTI related factors with severe injuries (n=425).

Variables	Unadjusted OR (95% CI)	Adjusted OR (95% CI)	p-value
***Age***			
4-17 (n=67)	1.00	1.00	
18-29 (n=183)	0.94 (0.42-2.07)	1.33 (0.52-3.38)	0.549
30-39 (n=86)	0.92 (0.37-2.29)	1.04 (0.36-2.93)	0.942
40 and above(n=89)	1.54(0.66-3.59)	1.58 (0.62-4.05)	0.343
***Education***			
Illiterate(n=107)	1.00	1.00	
primary(n=112)	0.83 (0.41-1.67)	0.99 (0.45-2.21)	0.991
Matric(n=125)	0.59 (0.28-1.22)	0.79 (0.35-1.75)	0.568
Inter& above (n=87)	0.90 (0.42-1.93)	1.16 (0.50-2.71)	0.719
***Vehicle involved in RTA***			
Others(n=53)	1.00	1.00	
Bike(n=372)	0.32 (0.17-0.62)	0.27 (0.12-0.59)	0.001
***Nature of RTA***			
Slip (n=154)	1.00	1.00	
Collision with other vehicle/thing (n=271)	2.69 (1.42-5.12)	3.16 (1.53-6.51)	0.002
***Time of RTA***			
Morning (n=112)	1.00	1.00	
Daytime (n=90)	1.01 (0.45-2.23)	1.04 (0.44-2.46)	0.921
Evening (n=94)	1.42(0.68-2.97)	1.31 (0.57-2.99)	0.523
Night (n=129)	1.10 (0.54-2.24)	1.10 (0.50-2.42)	0.808
***Site of RTA***			
Others(n=106)	1.00	1.00	0.270
Main road(n=312)	0.74 (0.41-1.32)	0.70 (0.37-1.31)	
***Victims.***			
Others (n=153)	1.00	1.00	0.389
Rider/driver(n=272)	0.93 (0.54-1.60)	1.33 (0.69-2.54)	
***Over-speeding***			
No	1.00	1.00	0.846
Yes	0.91 (0.52-1.60)	0.93 (0.48-1.79)	
***Wrong Way/Signal break***			
No	1.00	1.00	0.517
Yes	0.82 (0.30-2.19)	0.69 (0.23-2.06)	
***Sudden Break/Turn***			
No	1.00	1.00	0.682
Yes	0.77 (0.41-1.44)	0.86 (0.43-1.72)	
***Stuck-Clothing in wheels***			
No	1.00	1.00	0.974
Yes	0.43 (0.10-3.41)	1.03 (0.11-9.66)	
***Crossing blindly***			
No	1.00	1.00	0.037
Yes	4.73 (1.4-15.97)	4.34 (1.09-17.20)	
***Road Condition***			
Dry	1.00	1.00	0.009
Wet		3.96 (1.41-11.09)	

## DISCUSSION

In our research, two-thirds of RTI victims were men between the ages of 18 and 39 which is consistent with previous studies that report high occurrence of RTIs among males of productive age.^11^-^13^ Motorbikes were the most vulnerable to be involved in RTIs followed by rickshaws possibly due to their fragility associated with them being two and three wheelers. Previous studies from Pakistan also report a high vulnerability of motorbike riders to RTIs.^14^,^15^ More than half of RTIs were triggered by head-on vehicle-to-vehicle collisions which is consistent with a previous study from Maldives.^16^ RTIs mostly occurred on main roads and highways followed by link roads and residential streets since vehicles operate at higher speeds on main roads. This is in conformity with the results of a study in India.^17^ Our findings also indicated that RTIs were slightly higher at night time possibly due to poor visibility and inadequate street lighting.

We analyzed the cause of injuries in two groups, drivers/riders and pedestrian/passengers. Violations of traffic rules, over-speeding and neglecting the road laws were the predominant reasons reported by both. Similar reasons have been reported by Khan FM et al.^18^ A significant number of motorbike riders got entangled by long-clothing coiling the wheels. Another important trigger was careless crossing of roads by pedestrians.

The majority of injuries were minor, which is in accordance with a study from Lahore.^19^ A staggering 87.9% of bike riders ignored wearing helmets which is alarming as a previous study has reported a higher likelihood of severe injuries in those who did not wear helmet.^20^ Bikers, as compared to car drivers, had a lower chance of acquiring severe injuries because they are usually involved in sliding and gliding rather than a head-on collision. The serious injuries were more pronounced in incidents involving head-on collisions. Slippery roads and pedestrians crossing roads blindly were two reasons that showed a strong positive association with serious injuries. Although **some** intensity is reduced in the cars, but direct impact tends to have more detrimental injuries. Furthermore, wet roads in rainy conditions may worsen the head-on collisions.

### Limitations of the study:

The study was done on a small scale and conducted in one hospital. However, the data represent the biggest hospital in the city where almost half of the RTIs are managed. Second, the study is subject to response bias as it relied on the history provided by the victim. Third, the RTIs resulting in deaths were excluded from the study which would have underestimated the proportion of RTIs leading to severe injuries. Last, due to cross sectional nature of the study, reasons can only be identified and cause effect relationship cannot be proved.

## CONCLUSION

It was observed that motorbikes were involved in majority of RTIs. Some of the basic safety and preventive practices were grossly violated which included over-speeding, careless road crossing, breaking the signal and riding on wet roads which lead to moderate to severe injuries in almost two thirds of participants

### Recommendations:

Based on the findings, this study has a few recommendations. Educational and Regulatory interventions are needed to decrease the occurrence of RTIs and their potential consequences. Separate lanes for bike riders and strict compliance to ride on separate lanes can help in reducing the RTIs involving Bike Riders. Implementation of basic traffic laws is week which needs to be strengthened and people involved in negligent behavior of over-speeding, carelessly taking wrong ways or breaking the signal, careless road crossing, riding on wet roads and not taking care of long hanging clothes getting stuck in the wheel shall be strictly fined. We also found that RTIs were more frequent during night time and some events occurred to poor road infrastructure. This calls for an improved road infrastructure and street lighting system. Awareness campaigns in improving road behavior and enforcement of laws to curtail irresponsible behaviors shall be introduced. More research is required on RTIs to determine their impact on economy and quality of life.

### Author’s Contributions

**KM and SS** conceived, designed and did statistical analysis & editing of manuscript.

**JA and SH** did data collection and assisted in manuscript writing by reviewing the literature, developing the tables and writing the discussion

**KM** takes the responsibility and is accountable for all aspects of the work in ensuring that questions related to the accuracy or integrity of any part of the work are appropriately investigated and resolved.
